# Broad humoral and cellular immunity elicited by one-dose mRNA vaccination 18 months after SARS-CoV-2 infection

**DOI:** 10.1186/s12916-022-02383-4

**Published:** 2022-05-04

**Authors:** Chang Kyung Kang, Hyun Mu Shin, Pyoeng Gyun Choe, Jiyoung Park, Jisu Hong, Jung Seon Seo, Yung Hie Lee, Euijin Chang, Nam Joong Kim, Minji Kim, Yong-Woo Kim, Hang-Rae Kim, Chang-Han Lee, Jun-Young Seo, Wan Beom Park, Myoung-don Oh

**Affiliations:** 1 Department of Internal Medicine, Seoul National University College of Medicine, Seoul, 03080 South Korea; 2 Department of Biomedical Sciences, Seoul National University College of Medicine, Seoul, 03080 South Korea; 3 BK21 FOUR Biomedical Science Project, Seoul National University College of Medicine, Seoul, 03080 South Korea; 4 Wide River Institute of Immunology, Seoul National University, Hongcheon, 25159 South Korea; 5 Department of Pharmacology, Seoul National University College of Medicine, Seoul, 03080 South Korea; 6 Severance Biomedical Science Institute, Brain Korea 21 Project for Medical Science, Yonsei University College of Medicine, Seoul, 03722 South Korea; 7 Department of Internal Medicine, Seoul Asan Medical Center, Seoul, 05505 South Korea; 8 Department of Anatomy & Cell Biology, Seoul National University College of Medicine, Seoul, 03080 South Korea; 9 Medical Research Institute, Seoul National University College of Medicine, Seoul, 08030 South Korea

**Keywords:** SARS-CoV-2, COVID-19, Vaccination, Immune response, mRNA

## Abstract

**Background:**

Practical guidance is needed regarding the vaccination of coronavirus disease 2019 (COVID-19) convalescent individuals in resource-limited countries. It includes the number of vaccine doses that should be given to unvaccinated patients who experienced COVID-19 early in the pandemic.

**Methods:**

We recruited COVID-19 convalescent individuals who received one or two doses of an mRNA vaccine within 6 or around 18 months after a diagnosis of severe acute respiratory syndrome-coronavirus-2 (SARS-CoV-2) infection. Their samples were assessed for IgG-binding or neutralizing activity and cell-mediated immune responses against SARS-CoV-2 wild-type and variants of concern.

**Results:**

A total of 43 COVID-19 convalescent individuals were analyzed in the present study. The results showed that humoral and cellular immune responses against SARS-CoV-2 wild-type and variants of concern, including the Omicron variant, were comparable among patients vaccinated within 6 versus around 18 months. A second dose of vaccine did not significantly increase immune responses.

**Conclusion:**

One dose of mRNA vaccine should be considered sufficient to elicit a broad immune response even around 18 months after a COVID-19 diagnosis.

**Supplementary Information:**

The online version contains supplementary material available at 10.1186/s12916-022-02383-4.

## Background

Despite the repeated emergence and dissemination of new severe acute respiratory syndrome coronavirus-2 (SARS-CoV-2) variants of concern (VOCs), vaccination remains one of the most crucial measures to mitigate the coronavirus disease-2019 (COVID-19) pandemic [1]. SARS-CoV-2 vaccination is routinely recommended for patients who have recovered from COVID-19 [2], supported by immunologic studies showing that strong immune responses against SARS-CoV-2 are conferred by additional vaccination [3–5]. In addition, achieving a broad immune response covering VOCs is essential to reduce the risk of reinfection and severe COVID-19, particularly in the current era of variants. Several studies have reported broad humoral and cellular immunity with one dose of an mRNA vaccine in COVID-19 convalescent individuals [3, 6–8]. However, data on the optimal time window of vaccination after COVID-19 defined as that inducing a robust immune response are still lacking, since previous studies did not examine COVID-19 convalescents who had been vaccinated more than a year after the diagnosis of COVID-19.

Vaccination of COVID-19-convalescent individuals in resource-limited settings is challenging. By the end of December 2021, only 9% of the African population had been fully vaccinated, and the vaccination rate in convalescent individuals is probably even lower [9]. Under these conditions, practical guidance is needed regarding the additional vaccination of COVID-19 convalescent individuals, including the number of vaccine doses that should be given to unvaccinated patients who experienced COVID-19 early in the pandemic. Adequate vaccination of this group can be expected to play an essential role in controlling the pandemic by boosting immunity to SARS-CoV-2, mainly since new VOCs have been repeatedly emerging from vulnerable areas [10].

Therefore, in the present study, we compared the humoral and cellular immune responses against SARS-CoV-2 wild-type (WT) and VOCs between individuals who received one or two doses of an mRNA vaccine within 6 and around 18 months after a COVID-19 diagnosis.

## Methods

### Study design and participants

Serum and peripheral blood mononuclear cell (PBMC) samples were prospectively collected from COVID-19 convalescent individuals who received one dose of an mRNA vaccine either within 6 months (Conv6mVx1 group) or around 18 months (Conv18mVx1 group) of a COVID-19 diagnosis. Additional serum samples were collected from COVID-19 convalescent individuals who received two doses of an mRNA vaccine (Conv6mVx2 and Conv18mVx2 groups) and from healthy healthcare workers (NonConvVx0, NonConvVx1, and NonConvVx2 groups). The post-vaccination sample was collected 2−4 weeks after each vaccination. All serum samples were stored at −80 °C until use in the assays.

All serum samples were examined for IgG-binding activity against the receptor-binding domain (RBD) of SARS-CoV-2 WT and the Alpha, Beta, Delta, and Omicron variants (RBD_wt_, RBD_α_, RBD_β_, RBD_γ_, RBD_δ_, and RBD_ο_). Samples from the Conv6mVx1, Conv18mVx1, Conv18mVx2, NonConvVx1, and NonConvVx2 groups were additionally assessed for neutralizing activity, and PBMCs from the Conv6mVx1 and Conv18mVx1 groups were assessed for cell-mediated immune responses against WT SARS-CoV-2 and the Delta variant.

All COVID-19 convalescent individuals had been laboratory-confirmed with real-time reverse transcription polymerase chain reaction and admitted to the Seoul National University Hospital or a community treatment center and had mild or asymptomatic disease with no oxygen requirement [11, 12]. The Institutional Review Board of Seoul National University Hospital approved the study (IRB nos. 2009-168-1160 and 2102-032-1193). Written informed consent was obtained from all participants according to the Declaration of Helsinki.

### Preparation of recombination SARS-CoV-2 antigens

Genes encoding RBD of SARS-CoV-2 Alpha, Beta, Gamma, Delta, and Omicron variants were cloned in-frame into the pcDNA3.4-SARS-CoV-2-spike RBD-his using Gibson Assembly cloning (NEB, Ipswich, MA, USA) [13]. SARS-CoV-2 antigens were produced in Expi293 cells (Thermo Fisher Scientific) and his–tagged SARS-CoV-2 antigens protein was purified using Ni-NTA agarose resin (Thermo Fisher Scientific) affinity chromatography, as described previously [13].

After 5 days of transfection, the supernatant was collected and passed over the Ni-NTA agarose resin column three times. First, the column was washed with 100 mL of 1× PBS to remove nonspecific bound proteins. Then, 3 mL of elution buffer (pH8.0, 50 mM sodium phosphate, 300 mM NaCl, and 250 mM imidazole) was added to elute the bound proteins. Finally, samples were buffer-exchanged into pH 7.4 PBS using Amicon Ultra-4 (Merck Millipore, Burlington, MA, USA) spin columns with a 10 kDa cutoff. The purity of purified samples was assessed by 14% SDS-PAGE gel (Additional file 1: Supplementary Fig. 1).

### Binding antibody enzyme-linked immunosorbent assay

For measuring the binding activity of serum antibody (Ab) against each SARS-CoV-2 antigen (RBD_wt_, RBD_α_, RBD_β_, RBD_γ_, RBD_δ_, and RBD_ο_), 100 ng per well of each antigen was coated on a 96-well polystyrene enzyme-linked immunosorbent assay (ELISA) plate (Thermo Fisher Scientific) for overnight at 4 °C. After blocking with 1× PBS (pH 7.4) containing 3% bovine serum albumin (BSA) for 1 h at room temperature, the plate was washed four times with the PBST buffer (PBS with 0.05% Tween 20). The diluted plasma (1:50) was added and incubated at room temperature for 1 h.

After washing with the PBST buffer four times to detect IgG level, mouse anti-human IgG Fc Ab–conjugated with HRP (1:12,000, Arigobio, Hsinchu, Taiwan) was added and incubated for 1 h at room temperature. After washing four times with the PBST buffer, 50 μL of 3,3′,5,5′-tetramethylbenzidine substrate was added per well (Thermo Fisher Scientific) and then 50 μL of 2 M H_2_SO_4_ was added to neutralize. Finally, the absorbance at 450 nm was measured using the Infinite 200 PRO NanoQuant microplate readers (Tecan Trading AG, Männedorf, Switzerland).

### Cells and viruses

Vero E6 (ATCC# CRL-1586) cells were maintained in Dulbecco’s modified Eagle’s medium (DMEM) supplemented with 10% fetal bovine serum (FBS; Hyclone, South Logan, Utah, USA) and 1% antibiotics (Cytiva, South Logan, Utah, USA) [14]. SARS-CoV-2 isolates, SARS-CoV-2/human/KOR/KCDC03-NCCP 43326/2020 (GenBank ID. MW466791.1, S clade) [15], and SARS-CoV-2/human/KOR/NCCP 43390/2021 (GenBank ID. OL966962.1, Delta variant) were obtained from the National Culture Collection for Pathogens, Korea Disease Control, and Prevention Agency.

### Virus propagation

Vero E6 cells were inoculated with SARS-CoV-2 isolate at 1 multiplicity of infection (MOI), and fresh DMEM with 2% FBS and 1% antibiotics was added. At 48 h after infection, the supernatants of virus-infected cells were harvested and stored at −80 °C. Viral titers were determined by plaque assay on Vero E6 cells as described previously [16, 17]. All work with SARS-CoV-2 was conducted in biosafety level 3 facilities in accordance with the guidelines of and approved by the Institutional Biosafety Committee of Yonsei University Health System.

### Plaque assay

Vero E6 cells were seeded into 12-well plates at 24 h before infection. Cells were inoculated with ten-fold serial dilutions of SARS-CoV-2 samples and adsorbed at 37 °C in a 5% CO_2_ incubator for 1 h, rocking every 15 min. After virus adsorption, inoculums were removed from cells and then 1.5 ml of 1% agarose (Lonza 50101, Rockland, ME, USA) prepared in DMEM with 2% FBS and 1% antibiotics was added. To stain plaques performed with an agarose overlay, 4% paraformaldehyde (Biosesang, Gyeonggi-do, Republic of Korea) was added on top of the agarose and incubated for 24 h at 4 °C. The agarose plug was removed, and the fixed monolayer was stained with 0.5% crystal violet in 20% methanol. The monolayers were washed with topwater. The number of plaques was counted. Viral titers were calculated in plaque-forming units (PFU) per milliliter.

### Focus reduction neutralization test

The focus reduction neutralization test (FRNT) was performed by a slightly modified version of a method described previously [18, 19]. A day before viral infection, Vero E6 cells were seeded into 96-well tissue culture plates (1 × 10^4^ cells/well) and incubated at 37 °C for 24 h. Heat-inactivated serum samples were centrifuged at 16,000×*g* for 20 s at 4 °C. The serum specimens were serially diluted with serum-free-DMEM in 96-well U bottom plates and mixed with an equal volume of SARS-CoV-2 viral stock (600 PFU/well) following 1 h incubation at 37 °C with 5% CO_2_. Subsequently, the immune complexes formed by serum and SARS-CoV-2 were overlaid on top of Vero E6 cells and incubated at 37 °C with 5% CO_2_ for 1 h. After virus adsorption, the immune complexes were removed and replaced by DMEM with 1% methylcellulose (Sigma-Aldrich, Saint Louis, MO, USA) and 2% FBS. The infected cells were incubated at 37 °C with 5% CO_2_ for 17 h and then the media containing methylcellulose were washed out with PBS. The cells were fixed with 2% paraformaldehyde (Biosesang, Gyeonggi-do, Republic of Korea) at 4 °C for 24 h and washed out with PBS. The fixed cells were permeabilized with 0.1% saponin (Sigma-Aldrich) in PBS containing 0.1% BSA for 20 min at room temperature and incubated with a rabbit anti-SARS-CoV/CoV-2 nucleocapsid Ab (Sino Biological, Beijing, China) for 1 h at room temperature. Following washing with PBS, the cells were incubated with a goat anti-rabbit IgG HRP-conjugated secondary Ab for 1 h at room temperature. The cells were incubated with KPL SureBlue Peroxidase Substrate (Seracare, MA, USA) at room temperature and washed with PBS. The cell control (CC) with only cells and the virus control (VC) with virus and cells were set up inonach plate. The foci were visualized and analyzed using an ImmunoSpot reader (Cellular Technology Limited, Cleveland, USA). The percentage of neutralization was calculated as follows: [1 – (the number of foci for sample – the number of foci for CC)/(the number of foci for VC – the number of foci for CC)] × 100. The inhibitory concentration that neutralizes 50% (IC_50_) of SARS-CoV-2 infection was calculated using a 4-parameter nonlinear regression of GraphPad Prism 9 software.

### Pseudovirus neutralization assay

Pseudovirus expressing the SARS-CoV-2 S protein was produced as described previously [20]. HIV-1 NL4-3 ΔEnv Vpr Luciferase Reporter Vector from Dr. Nathaniel Landau [21, 22] and GP-pCAGGS with SARS-CoV-2 S were obtained through the NIH AIDS Reagent Program, Division of AIDS, NIAID, NIH. These plasmids were co-transfected into 293T cells. Forty-eight hours later, SARS-CoV-2 pseudovirus-containing supernatants were harvested, filtered through a 0.45-μm hydrophilic polyethersulfone syringe filter (Pall Corporation, Port Washington, NY, USA), and concentrated by ultracentrifugation at 25700 rpm for 3 h at 4 °C in a Beckman SW32Ti swinging bucket rotor lined with a Beckman thin wall polypropylene centrifuge tube (Beckman Coulter, High Wycombe, UK). The number of infectious virus particles was quantified using the Median Tissue Culture Infectious Dose (TCID_50_) assay [23]. 1300 TCID_50_/mL of pseudovirus was mixed with 6 serially diluted serum samples from the COVID-19 patients at 37 °C for 1h. Then, the mixtures were transferred to 96-well plates containing monolayers of 293T cells expressing ACE2 (hACE2-293T) (Takara Bio Inc., Japan). After incubation for 48 h, the cells were harvested with 100 μL of 1× luciferase cell culture lysis reagent and analyzed for luciferase activity by the addition of luciferase substrate using GloMax® discover (all from Promega, Madison, WA, USA). Percent neutralization was normalized as 100% neutralization of uninfected cells and 0% neutralization of pseudovirus-only infected cells.

### Collection of PBMCs and antigen stimulation

The whole blood was collected in heparin-containing Vacutainer tubes (Becton Dickinson). PBMCs were purified after blood collection using Ficoll-Hypaque (1.077 g/mL; GE Healthcare Life Sciences, Piscataway, NJ, USA). The PBMCs were then stored in liquid nitrogen in a serum-free cryopreservation medium (Cellbanker 2, Zenoaq) until examined [24]. Cells were cultured in complete RPMI-1640 containing 10% FBS and 1× penicillin/streptomycin (Thermo Fisher Scientific) and stimulated as follows.

After thawing, 1×10^6^ PBMCs/mL were immediately stimulated with 0.06 nmol/mL of PepTivator® SARS-CoV-2 Prot_S Complete, PepTivator® SARS-CoV-2 Prot_S B.1.617.2 WT reference, or PepTivator® SARS-CoV-2 Prot_S B.1.617.2 Mutation Pool (all from Miltenyi Biotec, Bergisch Gladbach, Germany) for 24 h. A CEF peptide pool (Mabtech AB, Hamburg, Germany) and medium alone were used as a positive and negative control. Anti-human CD28/CD49d Abs for co-stimulation (clone L293/L25) and Brilliant Blue 515–anti-human CD4 (clone RPA-T4) Ab were added concomitantly with the antigens. Cells were treated with BD GolgiStop® (monensin) and BD GolgiPlug® (brefeldin A) for the final 4 h of the antigen stimulation.

### Fluorescence staining and flow cytometric analysis

After stimulation, dead cells were stained with LIVE/DEAD (Thermo Fisher Scientific). Cells were permeabilized and incubated with Brilliant Violet (BV) 510–anti-human CD3 (clone CHT1), peridinin chlorophyll protein complex–anti-human CD8 (clone SK1), phycoerythrin–indotricarbocyanine (Cy7)–anti-human interferon-γ (IFN-γ, clone B27), allophycocyanin–anti-human interleukin-2 (IL-2) (clone 5344.111), phycoerythrin–anti-human tumor necrosis factor-α (TNF-α) (clone Mab11), BV421–anti-human IL-4 (clone MP4-25D2), BV605–anti-human CD69 (clone FN50), and BUV395–anti-human CD137 (clone 4B4-1) Abs (all from BD Biosciences). BD Horizon Brilliant Stain Buffer (BD Biosciences) was added to each sample. Unstimulated cells and compensation beads (UltraComp eBeads, Thermo Fisher Scientific) were used in every experiment for compensation. Flow cytometry was performed using the FACSymphony (BD Biosciences) with a target event count of 1,000,000 cells. The flow cytometry results were analyzed using FlowJo software (version 10.7.1; TreeStar). A representative gating strategy is shown in Additional file 2: Supplementary Fig. 2.

To account for nonspecific cytokine production, the percentage of the target population in the unstimulated specimens was subtracted from the percentage in the stimulated cells [25]. The frequencies of SARS-CoV-2-specific activation-induced marker^+^ (AIM^+^) T cells (CD69^+^CD137^+^ CD4^+^ T cells or CD8^+^ T cells) [26] or cytokine-producing cells (among CD137^+^CD4^+^ or CD137^+^CD8^+^ T cells) were evaluated.

### Statistical analyses

The data are presented as the mean ± standard error of the mean (SEM) and as dot plots. A Wilcoxon signed-rank test (paired samples from uninfected healthcare workers) or Mann-Whitney *U* test (unpaired samples from COVID-19 convalescent individuals) was performed to compare immune responses between two groups. The Benjamini-Hochberg method was used to account for multiple comparisons between uninfected and infected groups. *P* < 0.05 was considered indicative of statistical significance. All statistical analyses were two-tailed and performed using GraphPad Prism 9 (GraphPad Software Inc., La Jolla, CA, USA). All graphs were drawn using GraphPad Prism 9.

## Results

### Study participants

We enrolled 10, 5, 18, and 10 individuals in the Conv6mVx1, Conv6mVx2, Conv18mVx1, and Conv18mVx2 groups, respectively. In addition, serum samples were collected from 10 uninfected healthcare workers in the NonConvVx groups. All COVID-19-convalescent individuals had a SARS-CoV-2 infection before the Delta surge in Korea [27].

The median age was 45, 43, 27, 30, and 33 years in the Conv6mVx1, Conv6mVx2, Conv18mVx1, Conv18mVx2, and NonConvVx groups, respectively. None of the participants had significant concurrent medical conditions. Among the convalescent individuals, none had received specific treatment for COVID-19, and none had a known history of re-exposure to SARS-CoV-2 after the initial infection. Detailed information on the type of mRNA vaccine, the interval between the COVID-19 diagnosis and vaccination, and the interval between each vaccination and sample collection are reported in Additional file 3: Supplementary Table 1.

### IgG-binding activities against SARS-CoV-2 WT and VOCs

**Fig. 1:**
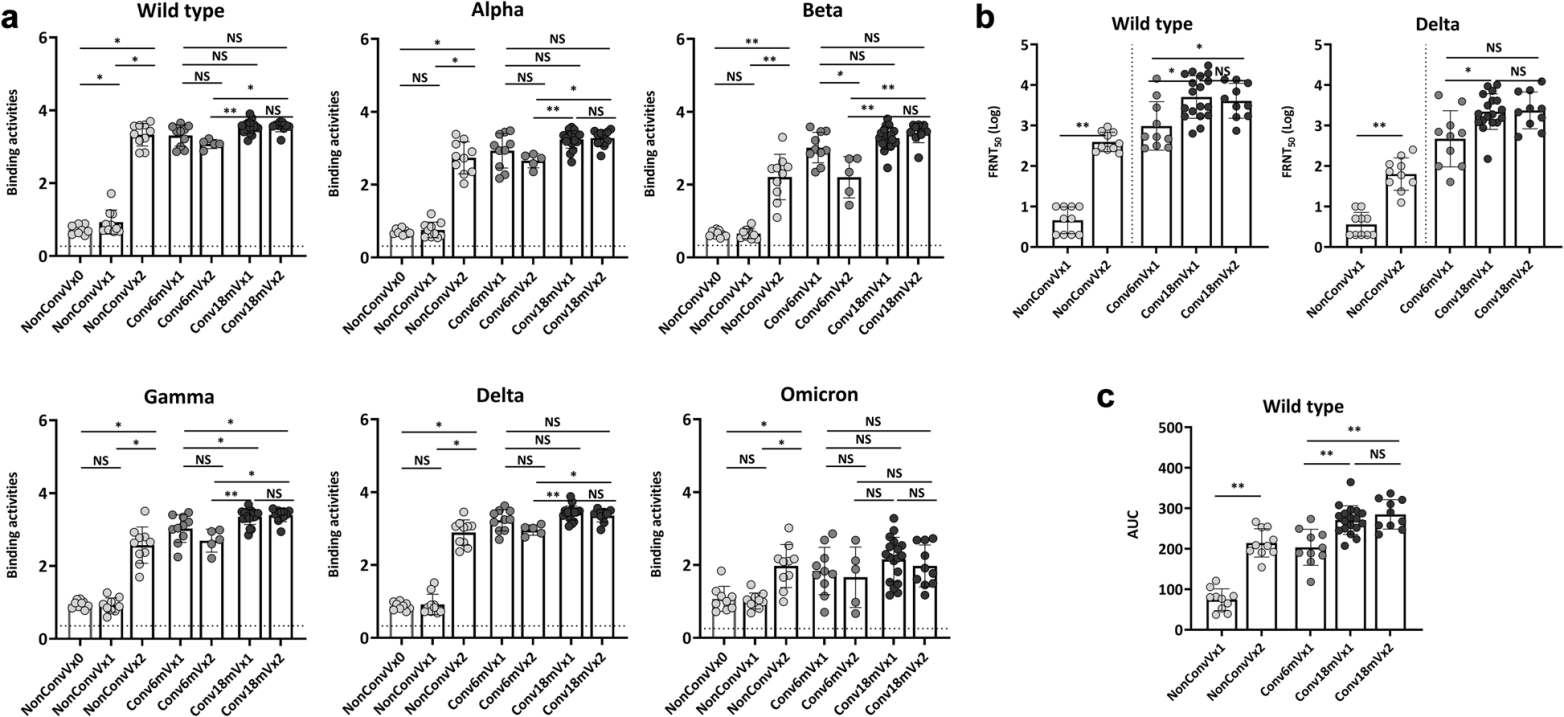
Humoral responses against different strains of SARS-CoV-2 according to vaccination timing and doses after mild COVID-19. **a** IgG-binding activities measured using an enzyme-linked immunosorbent assay. **b** Neutralizing activities measured using a live virus neutralization test. **c** Neutralizing activities based on a pseudovirus neutralization test. Horizontal lines in **a** denote binding activity values from the negative control specimen. *P < 0.05, **P < 0.005 NS, not significant; FRNT, focus reduction neutralization test; AUC, area under the curve

All serum samples were examined for serum IgG binding activity using ELISA against the RBD of SARS-CoV-2 WT (RBD_wt_), Alpha (RBD_α_), Beta (RBD_β_), Gamma (RBD_γ_), Delta (RBD_δ_), and Omicron (RBD_ο_) variants. Although the NonConvVx group had a significant humoral response by the second vaccination, humoral responses in the Conv6mVx and Conv18mVx groups were readily boosted by the first vaccination (Fig. 1a).

Serum IgG of Conv18mVx1 showed equivalent binding activities for RBD_wt_, RBD_α_, RBD_β_, RBD_δ_, and RBD_ο_ comparing with those of the Conv6mVx1 groups (Fig. 1a). The serum IgG RBD_γ_-binding activities were significantly stronger in the Conv18mVx1 group (median [interquartile range, IQR], 3.39 [3.30−3.48]) than in the Conv6mVx1 group (median [IQR], 3.07 [2.77−3.36]) (adjusted *P* = 0.047). Interestingly, the second vaccination did not improve the humoral IgG response to any of the RBD variants, either in the Conv6mVx or Conv18mVx group, and the response to the Beta variant declined (median [IQR], 2.95 [2.75−3.46] in the Conv6mVx1 group vs. 2.34 [1.62−2.71] in the Conv6mVx2 group, adjusted *P* = 0.032). The binding activity of serum IgG from the Conv18mVx1 group was significantly lower for RBD_ο_ than the other RBD variants (Additional file 4: Supplementary Fig. 3a), as previously reported [28].

### Neutralizing activities against SARS-CoV-2 WT or Delta variant

Neutralizing activity against SARS-CoV-2 WT and the Delta variant was investigated in FRNT_50_ using serum samples from the Conv6mVx1, Conv18mVx1, Conv18mVx2, NonConvVx1, and NonConvVx2 groups. Log-transformed FRNT_50_ values were higher in the Conv18mVx1 than the Conv6mVx1 group (median [IQR] for WT, 2.72 [2.49−3.52] in the Conv6mVx1 group vs. 3.66 [3.25−4.24] in the Conv18mVx1 group, adjusted *P* = 0.013; for the Delta variant, 2.78 [1.99−3.15] in the Conv6mVx1 group vs. 3.29 [3.08−3.66] in the Conv18mVx1 group, adjusted *P* = 0.013) (Fig. 1b). A pseudovirus neutralization test against WT SARS-CoV-2 yielded similar results, where area under the curve (AUC) values were higher in the Conv18mVx1 than the Conv6mVx1 group (median [IQR], 196.40 [179.63−238.30] in the Conv6mVx1 group vs. 271.50 [248.88−289.15] in the Conv18mVx1 group, adjusted *P* < 0.001) (Fig. 1c).

Similar to the results of the serum IgG-binding activity assay, the second dose of an mRNA vaccine did not induce an additional response in the Conv18mVx group. Still, it significantly raised the FRNT_50_ and AUC values in the uninfected group. The neutralizing activity of the serum samples in the Conv18mVx1 group, as measured in a live virus neutralization assay, was considerably lower against the Delta variant than against WT SARS-CoV-2 (Additional file 4: Supplementary Fig. 3b)

### Cell-mediated immune responses against SARS-CoV-2 WT and the Delta variant

T cell-mediated immune responses against SARS-CoV-2 WT, and the Delta variant in the Conv6mVx1 and Conv18mVx1 groups were assessed using flow cytometric analysis. PBMCs were separately stimulated by three different SARS-CoV-2 antigens to examine the response to the whole spike peptide pool and matched WT and Delta spike mutation peptide pools. T cell-mediated immune responses were measured by the expression by AIM (CD69^+^CD137^+^ in both CD4^+^ and CD8^+^ T cells) or cytokines (IL-2, IL-4, TNF-α, and IFN-γ in both CD137^+^CD4^+^ and CD137^+^CD8^+^ T cells.

**Fig. 2:**
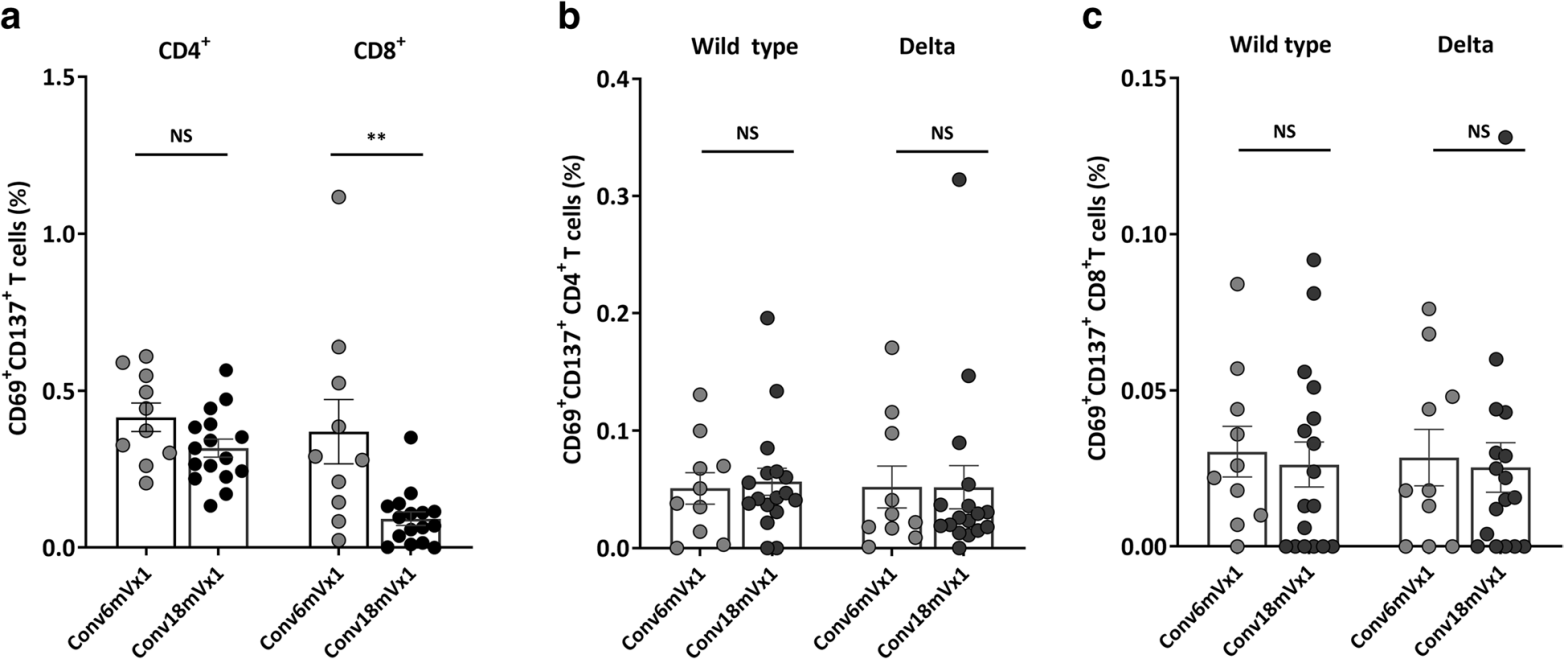
T cell-mediated immune responses against wild-type and Delta variant SARS-CoV-2 according to vaccination timing after COVID-19. **a** Activation-induced marker^+^ (AIM^+^) T cells stimulated by wild-type whole spike protein. **b**, **c** AIM^+^ CD4^+^**b** and CD8^+^**c** T cells stimulated by matched wild-type and Delta variant spike peptide pools. *P < 0.05, **P < 0.005 NS, not significant

The proportions of AIM^+^ CD4^+^ T cells against WT whole spike protein did not differ between the Conv6mVx1 and Conv18mVx1 groups (Fig. 2a), while those of CD8^+^ T cells were significantly higher in the Conv6mVx1 than in the Conv18mVx1 group (median [IQR], 0.29% [0.13−0.55%] vs. 0.08% [0.02−0.13%], *P* = 0.003). No significant differences were observed in the proportions of AIM^+^ T cells against matched WT and Delta mutation peptide pools (Fig. 2b, c).

The proportions of cytokine (IL-2, IL-4, TNF-α, or IFN-γ)-producing CD4^+^ or CD8^+^ T cells in the Conv6mVx1 and Conv18mVx1 groups also showed no significant difference (Additional file 5: Supplementary Fig. 4a and 4b). In addition, there was no significant difference in the proportions of AIM^+^ CD4^+^ or CD8^+^ T cells against WT and the Delta variant from the Conv18mVx1 group (Additional file 4: Supplementary Fig. 3c), as previously reported in the literature [6].

## Discussion

The magnitude and breadth of the humoral and cellular immune responses were generally similar between individuals who received one dose of an mRNA vaccine either within 6 months or around 18 months after their COVID-19 diagnosis. Indeed, serum IgG-binding activity against the Gamma variant and neutralizing activity against the WT and Delta variant were even higher in the Conv18mVx1 than in the Conv6mVx1 group. Moreover, the second dose of mRNA vaccine did not enhance immune responses, even in those vaccinated around 18 months after a COVID-19 diagnosis. This result implies that one dose of mRNA vaccine can be recommended long after recovery from COVID-19. The demonstrated immunogenicity of a single vaccine in this group is especially relevant in resource-limited countries, where many COVID-19 convalescent individuals have yet to be vaccinated.

Although the humoral and cellular immune responses were generally similar between our Conv6mVx1 and Conv18mVx1 groups, there were also several differences between these groups. Although the memory CD8^+^ T cell response after COVID-19 has declined over time and is generally lower than the memory CD4^+^ T cell response [29, 30], the proportion of AIM^+^ CD8^+^ T cells in our Conv18mVx1 group was particularly deficient in our study. On the contrary, serum IgG-binding activity against the Gamma variant, and neutralizing activity against the WT and Delta variant, were higher in the Conv18mVx1 than in the Conv6mVx1 group. These findings suggest that 18 months after the infection might be a lengthy time to optimally boost memory CD8^+^ T cell response, while memory B cell response could be boosted at the time frame.

Additional vaccination in COVID-19 convalescent individuals is recommended by the Centers for Disease Control and Prevention (CDC) [2], based on epidemiological observations of a significantly lower reinfection risk in COVID-19 patients who were subsequently vaccinated than in those who remained unvaccinated after infection [31]. The CDC’s recommendation is also supported by immunological findings of similar, or even greater, humoral and cellular responses elicited by one vaccine dose in patients who recovered from COVID-19 than by a two-dose vaccination regimen in naïve individuals [3–5, 8, 32]. Those studies found that a second dose did not significantly increase the immune response of COVID-19 convalescent individuals. However, there are no specific guidelines regarding the number of additional vaccine doses that should be given to people with a history of COVID-19, partly owing to a lack of knowledge of how long the maximal post-COVID-19 “boosting” effect could be generated by one dose of mRNA vaccine. We hypothesized that it could be achieved even after a year after COVID-19 diagnosis, and the present study results support the use of one additional vaccine dose, even in individuals who had COVID-19 as long as 18 months ago.

The SARS-CoV-2 vaccination coverage rate is still very low in resource-limited areas such as Africa [9]. The World Health Organization’s target vaccination rate of 40% by 2021 was achieved in only seven African countries. Our results indicate that a single vaccination of COVID-19 convalescent individuals in such areas of the world, even those whose recovery was more than 1 year ago, would help control the pandemic by inducing broad immunity against SARS-CoV-2 and thus suppressing the emergence of new VOCs.

Unexpectedly, IgG-binding activities were generally lower in the Conv6mVx2 than Conv6mVx1 group, although not significantly, with the exception of activity against the Beta variant. Since numerous studies have shown similar immune responses between after the first and second dose of vaccination [3–5], the results of our study may be partly due to the small sample size and the older age of participants in the Conv6mVx2 group. However, Lozano-Ojalvo et al. also reported a significant decline of SARS-CoV-2 peptide-pool-stimulated plasma IFN-γ levels after the second vaccination in people with a history of COVID-19 [32]. Since this could be involved in issues on immunological imprinting [33, 34], and would seem to call into question the value of a second additional vaccination, further studies are warranted.

The present study provides further evidence of partial humoral escape of VOCs, especially the Omicron variant [6]. By contrast, cell-mediated immune responses against the WT and delta variant RBD, as measured by the proportions of AIM^+^ T cells, were not statistically different in the present study [6]. Since a vaccine-elicited cross-reactive cell-mediated immune response against the Omicron variant has been reported [35] and might contribute to a less severe disease course following SARS-CoV-2 infection [36], the importance of vaccination could not be overemphasized.

The present study had several limitations. First, the sample size was small, and only nonconsecutive COVID-19 convalescent individuals could be enrolled. Second, other than IgG-binding activity, the immune response against the Omicron variant could not be evaluated. However, trends similar to those of the Delta variant are likely, based on extrapolation of the neutralizing activity and cellular immune responses against the Delta variant. Third, all of the COVID-19 convalescent individuals in this study had the mild illness. However, a boosting effect in those with severe disease can be assumed since immune memory response to SARS-CoV-2 is generally stronger in patients with severe than mild disease [29, 37]. Lastly, pre-vaccination samples could not be collected from convalescent individuals. However, given the well-documented decline in humoral and cellular responses after SARS-CoV-2 infection [38–40], the immune responses measured in the Conv6mVx and Conv18mVx groups were almost certainly elicited by mRNA vaccination.

## Conclusions

The humoral and cellular immune responses achieved by one dose of vaccination were comparable between individuals who received one dose of mRNA vaccine once within 6 months and around 18 months after SARS-CoV-2 infection. One dose of vaccination should be considered sufficient to confer a broad immune response, especially in the era of VOCs, to convalescent individuals with a history of COVID-19 of up to 18 months.

## Supplementary Information

**Additional file 1: Figure S1**. Purification of recombinant RBD proteins of SARS-CoV-2 variants. The purity of the purified recombinant protein was determined by non-reducing (left) and reducing (right) 14% SDS-PAGE gels. M, protein ladder. 1. RBD wild-type; 2. RBD Alpha (B.1.1.7, with N501Y mutation); 3. RBD Beta (B.1.351, with K417N, E484K, and N501Y); 4. RBD Gamma (P.1, with K417T, E484K, and N501Y mutations); 5. RBD Delta (B.1.617.2, with L452R and T478K mutations); 6. RBD Omicron (B. 1.1.529, with G339D, S371L, S373P, S375F, K417N, N440K, G446S, S477N, T478K, E484A, Q493R, G496S, Q498R, N501Y, and Y505H).

**Additional file 2: Figure S2**. Representative gating strategy for the SARS-CoV-2-specific activated and cytokine-producing T cells.

**Additional file 3: Table S1**. Detailed clinical information of each group in the present study

**Additional file 4: Figure S3**. Comparisons of humoral and cellular immune responses against different strains of SARS-CoV-2 in individuals who recovered and then received one dose of an mRNA vaccine 18 months later. **a** IgG-binding activities measured using an enzyme-linked immunosorbent assay. **b** Neutralization activities measured using a focus reduction neutralization test. **c** Proportions of activation-induced marker+ T cells measured by flow cytometric analysis. For simplicity, in Figure S3a, statistical significance is shown by the lines. * *P* < 0.05, ** *P* < 0.005 NS, not significant; FRNT, focus reduction neutralizing test.

**Additional file 5: Figure S4**. Cytokine-producing T cells against wild-type SARS-CoV-2 and the Delta variant according to vaccination status after COVID-19. **a** CD4+ T-cell populations producing specific cytokines. **b** CD8+ T-cell populations producing specific cytokines. NS, not significant; IFN-γ, interferon- γ; IL, interleukin; TNF-α, tumor necrosis factor-α.

## Data Availability

Correspondence and requests for data should be addressed to C.-H.L. (chlee-antibody@snu.ac.kr), J.-Y.S. (jyseo0724@yuhs.ac), or W.B.P. (wbpark1@snu.ac.kr). The individual participant data that underlie the results reported in this article will be shared after de-identification. The raw data will be available for 1 year after the publication of this article.

## Abbreviations

SARS-CoV-2: Severe acute respiratory syndrome-coronavirus-2
VOC: Variant of concern
COVID-19: Coronavirus disease-2019
WT: Wild-type
PBMC: Peripheral blood mononuclear cells
Conv6mVx1: The vaccinated once within 6 months after COVID-19
Conv18mVx1: The vaccinated once around 18 months after COVID-19
Conv6mVx2: The vaccinated twice within 6 months after COVID-19
Conv18mVx2: The vaccinated twice after 18 months around COVID-19
NonConvVx0: The uninfected and before vaccination
NonConvVx1: The uninfected and vaccinated once
NonConvVx2: The uninfected and vaccinated twice
RBD: Receptor-binding domain
Ab: Antibody
ELISA: Enzyme-linked immunosorbent assay
DMEM: Dulbecco’s modified Eagle’s medium
MOI: Multiplicity of infection
PFU: Plaque-forming units
FRNT: Focus reduction neutralizing test
CC: Cell control
VC: Virus control
IC_50_: The inhibitory concentration that neutralizes 50% of SARS-CoV-2 infection
TCID50: Median tissue culture infectious dose
BV: Brilliant violet
Cy7: Indotricarbocyanine
IFN-γ: Interferon-γ
IL-2: Interleukin-2
TNF-α: Tumor necrosis factor-α
AIM: Activation-induced marker
SEM: Standard error of the mean
IQR: Interquartile range
AUC: Area under the curve
CDC: Centers for Disease Control and Prevention
