# Supplementary figures and images for: Broad humoral and cellular immunity elicited by one-dose mRNA vaccination 18 months after SARS-CoV-2 infection
Source: BMC Med. 2022 May 4;20:181. doi: 10.1186/s12916-022-02383-4 (PMC9067342; doi:10.1186/s12916-022-02383-4)

Figure S1

Non-reducing SDS-PAGE (14%)

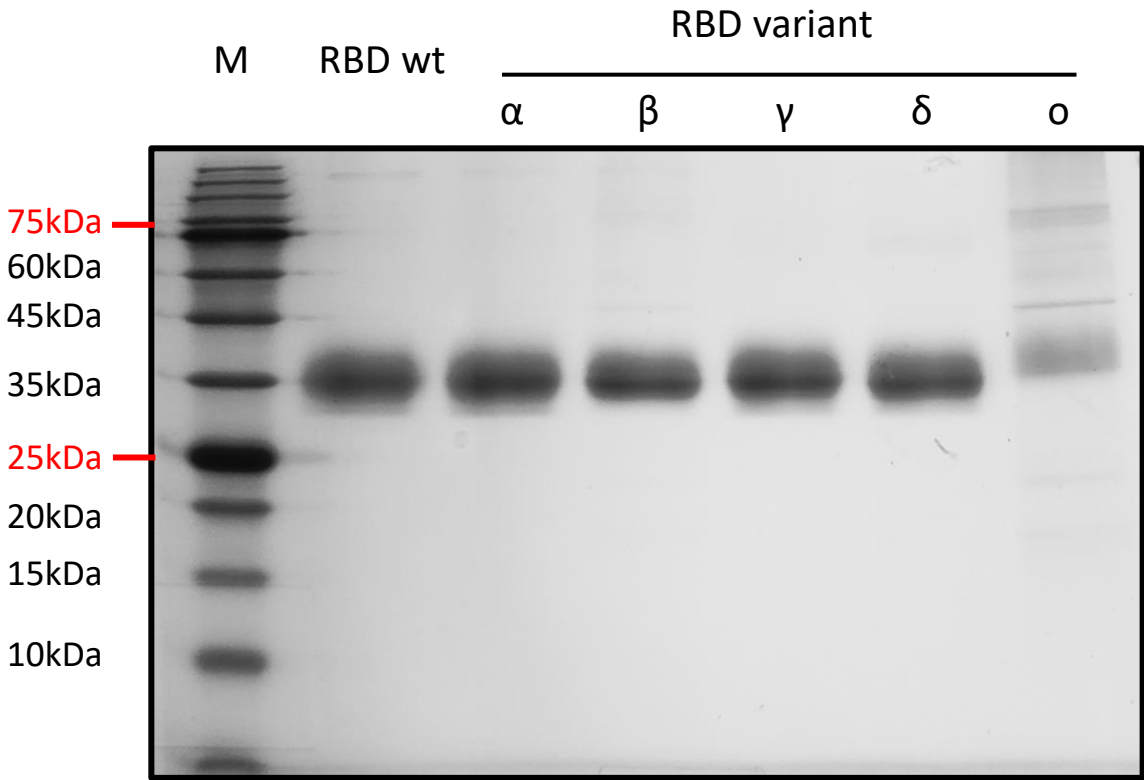

Reducing SDS-PAGE (14%)

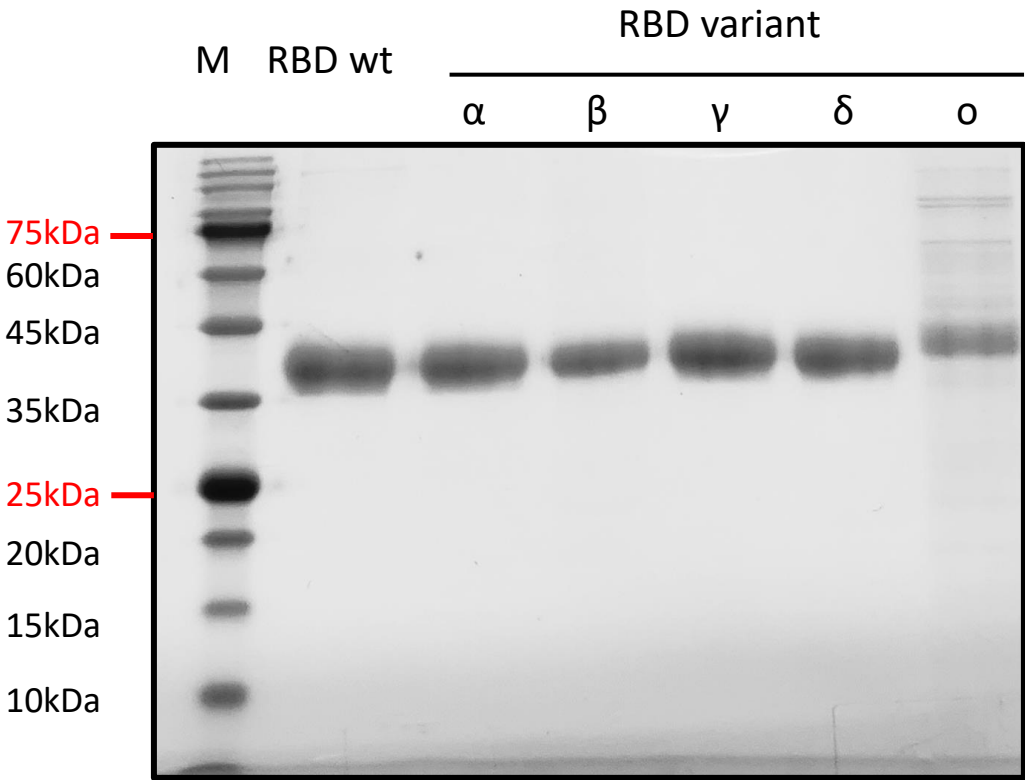

Supplement: Supplementary file 1 — Additional file 1: Figure S1. Purification of recombinant RBD proteins of SARS-CoV-2 variants. The purity of the purified recombinant protein was determined by non-reducing (left) and reducing (right) 14% SDS-PAGE gels. M, protein ladder. 1. RBD wild-type; 2. RBD Alpha (B.1.1.7, with N501Y mutation); 3. RBD Beta (B.1.351, with K417N, E484K, and N501Y); 4. RBD Gamma (P.1, with K417T, E484K, and N501Y mutations); 5. RBD Delta (B.1.617.2, with L452R and T478K mutations); 6. RBD Omicron (B. 1.1.529, with G339D, S371L, S373P, S375F, K417N, N440K, G446S, S477N, T478K, E484A, Q493R, G496S, Q498R, N501Y, and Y505H). [file 12916_2022_2383_MOESM1_ESM.pdf]

Figure S2

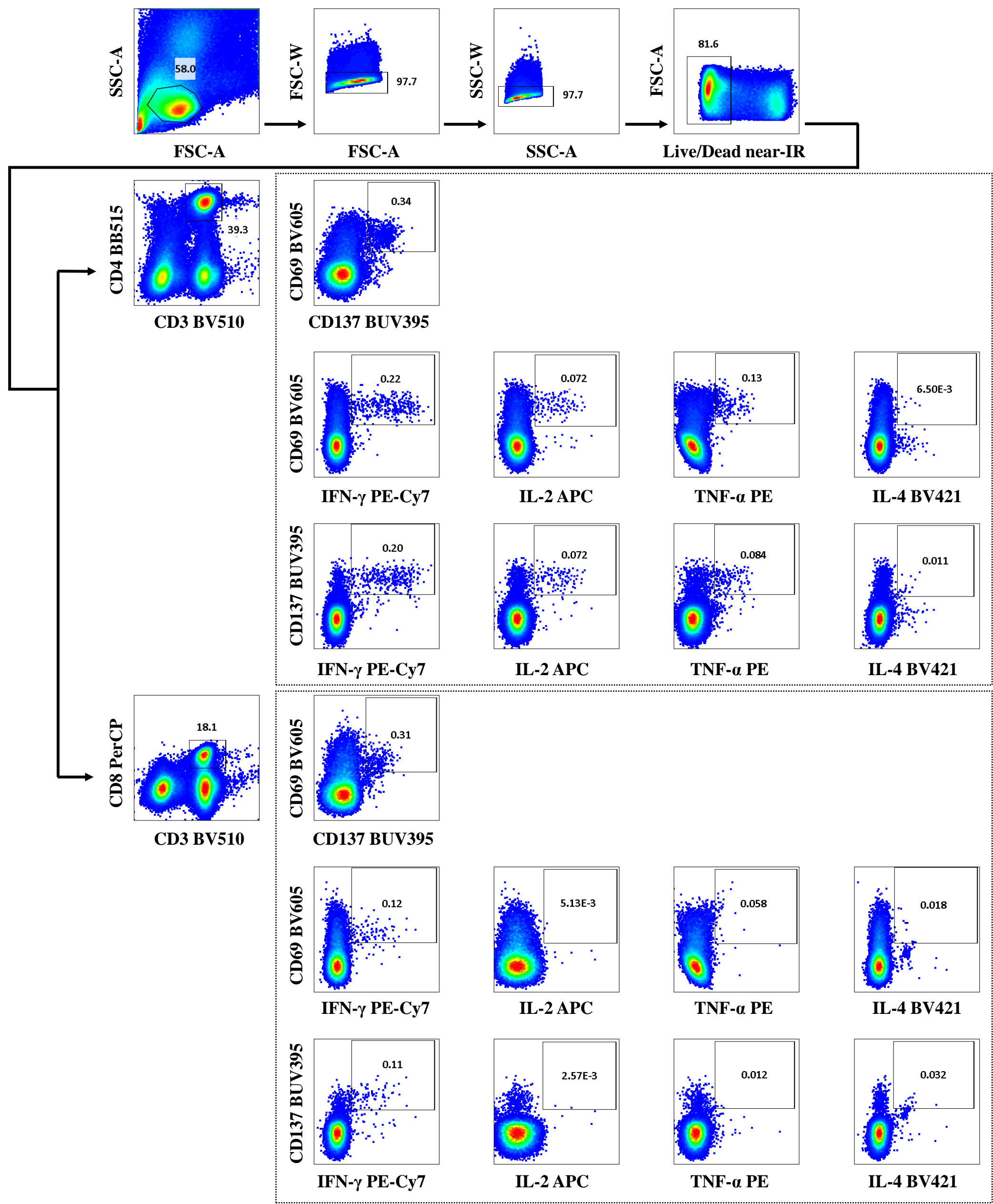

Supplement: Supplementary file 2 — Additional file 2: Figure S2. Representative gating strategy for the SARS-CoV-2-specific activated and cytokine-producing T cells. [file 12916_2022_2383_MOESM2_ESM.pdf]

Figure S3

a

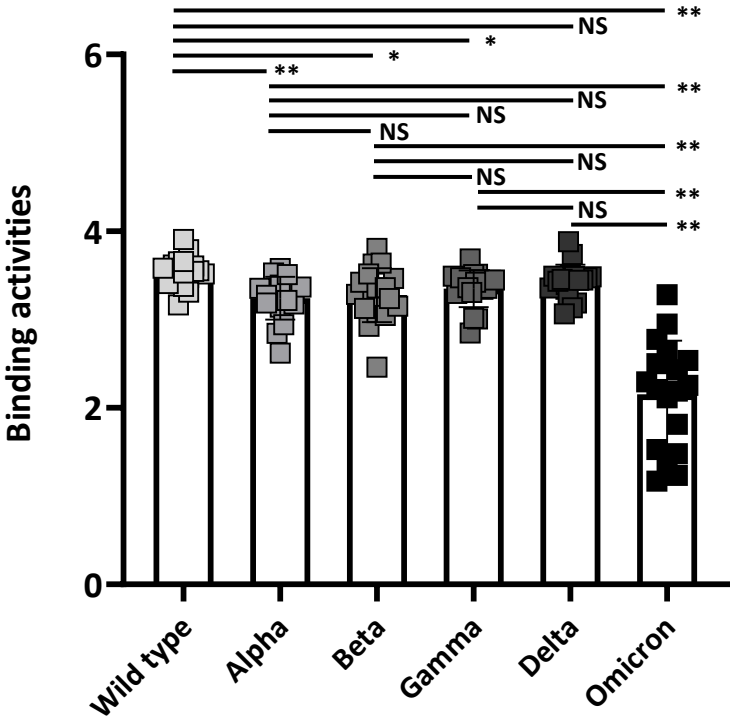

b

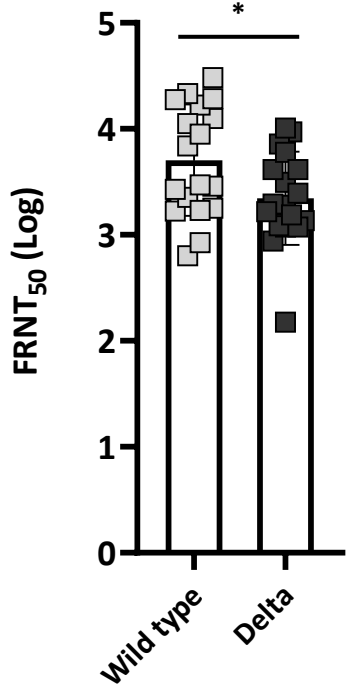

c

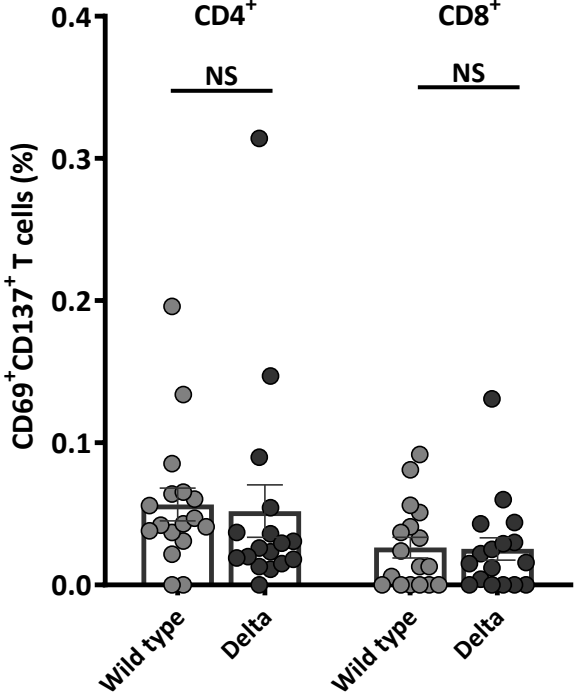

Supplement: Supplementary file 4 — Additional file 4: Figure S3. Comparisons of humoral and cellular immune responses against different strains of SARS-CoV-2 in individuals who recovered and then received one dose of an mRNA vaccine 18 months later. a IgG-binding activities measured using an enzyme-linked immunosorbent assay. b Neutralization activities measured using a focus reduction neutralization test. c Proportions of activation-induced marker+ T cells measured by flow cytometric analysis. For simplicity, in Figure S3a, statistical significance is shown by the lines. * P < 0.05, ** P < 0.005 NS, not significant; FRNT, focus reduction neutralizing test. [file 12916_2022_2383_MOESM4_ESM.pdf]

# Figure S4

**a**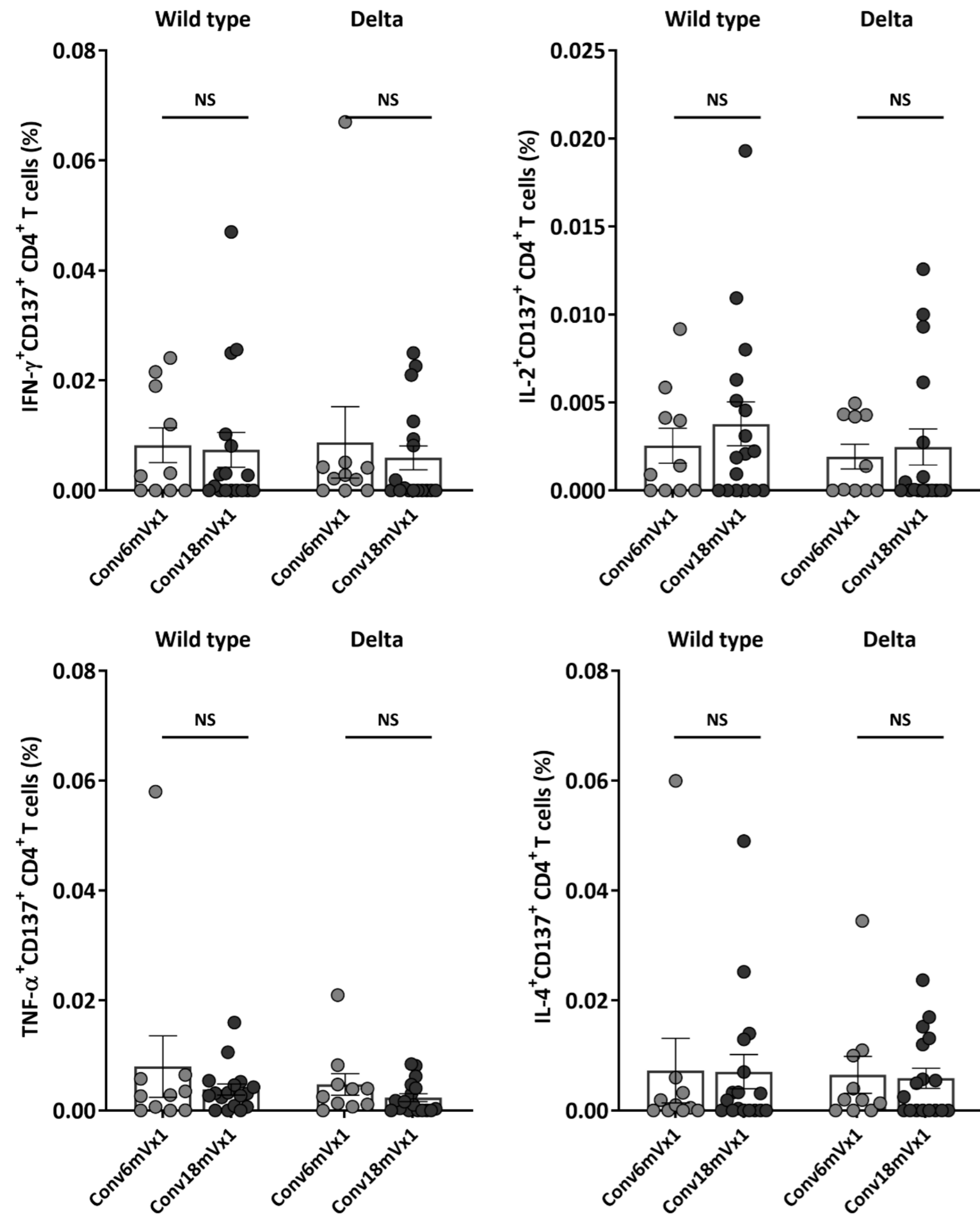**b**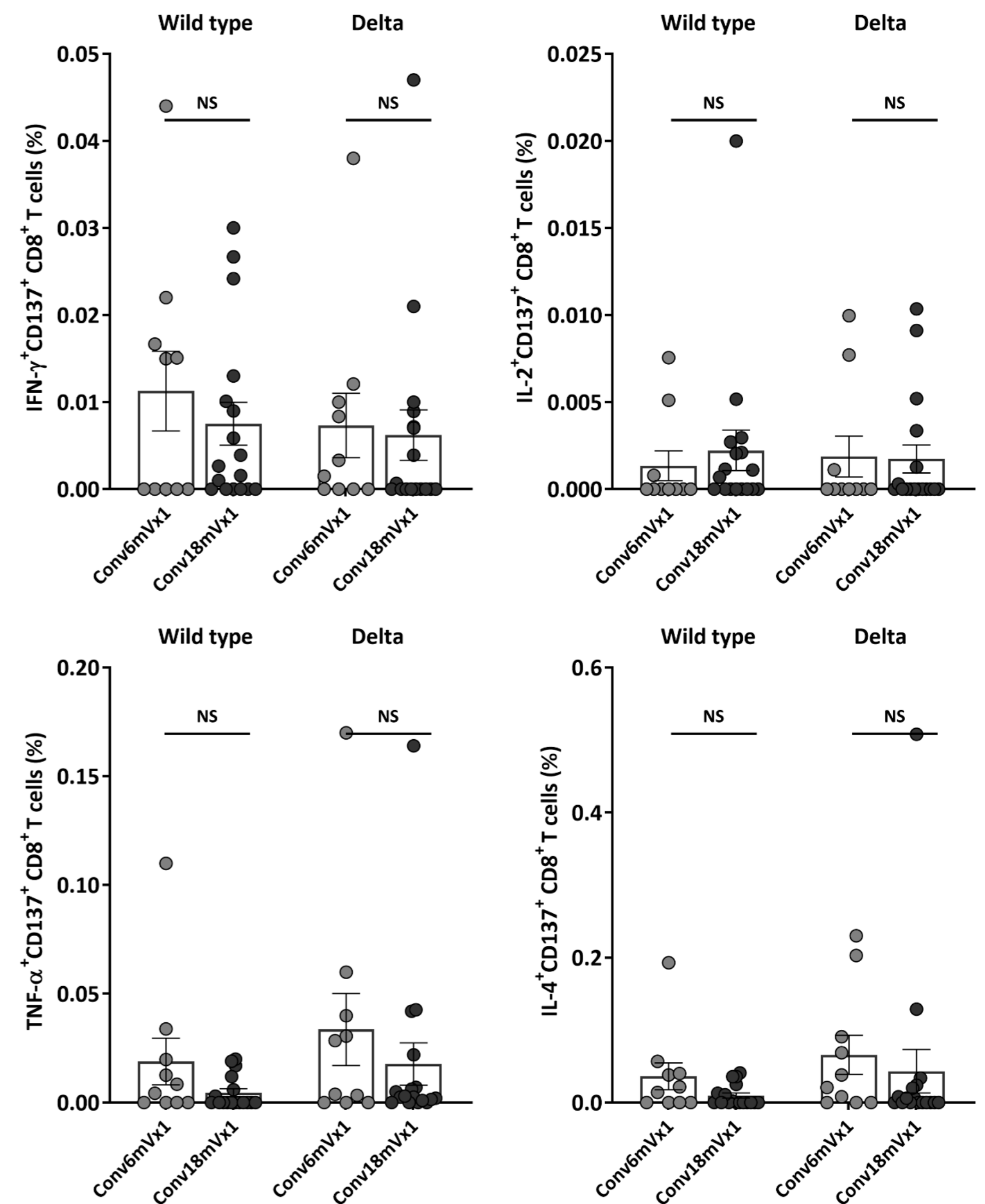

Supplement: Supplementary file 5 — Additional file 5: Figure S4. Cytokine-producing T cells against wild-type SARS-CoV-2 and the Delta variant according to vaccination status after COVID-19. a CD4+ T-cell populations producing specific cytokines. b CD8+ T-cell populations producing specific cytokines. NS, not significant; IFN-γ, interferon- γ; IL, interleukin; TNF-α, tumor necrosis factor-α. [file 12916_2022_2383_MOESM5_ESM.pdf]
