# Supplementary material for: Broad humoral and cellular immunity elicited by one-dose mRNA vaccination 18 months after SARS-CoV-2 infection
Source: BMC Med. 2022 May 4;20:181. doi: 10.1186/s12916-022-02383-4 (PMC9067342; doi:10.1186/s12916-022-02383-4)
Supplement: Supplementary file 3 — Additional file 3: Table S1. Detailed clinical information of each group in the present study [file 12916_2022_2383_MOESM3_ESM.pdf]

Table S1. Detailed clinical information of each group in the present study

	NonConvVx (n = 10)	Conv6mVx1 (n = 10)	Conv6mVx2 (n = 5)	Conv18mVx1 (n = 18)	Conv18mVx2 (n = 10)
Age, years, median (range)	33 (25−47)	45 (27−63)	43 (27−63)	27 (20−47)	30 (22−51)
Male sex, n (%)	5 (50)	4 (40)	1 (20)	5 (28)	4 (40)
Vaccine type					
BNT162b2, n (%)	10 (100)	10 (100)	5 (100)	6 (33)	6 (60)
mRNA-1273, n (%)	0 (0)	0 (0)	0 (0)	12 (67)	4 (40)
Days from the diagnosis of COVID-19 to 1st vaccination, median (IQR)	-	80 (45−146)	116 (51−176)	561 (558−570)	533 (529−539)
Days from vaccination to the sample collection, median (IQR)	NonConvVx1: 8 (7−10)<br>NonConvVx2: 9 (8−9)	32 (13−43)	27 (21−28)	26 (17−28)	12 (7−19)

NonConvVx, the uninfected and vaccinated group; Conv6mVx1, vaccinated once within 6 months after COVID-19; Conv6mVx2, vaccinated twice within 6 months after COVID-19; Conv18mVx1, vaccinated once around 18 months after COVID-19; Conv18mVx2, vaccinated twice after 18 months around COVID-19; IQR, interquartile range
